# Effect of Dried Distillers Grains With Solubles and Red Osier Dogwood Extract on Fermentation Pattern and Microbial Profiles of a High-Grain Diet in an Artificial Rumen System

**DOI:** 10.3389/fvets.2021.644738

**Published:** 2021-04-09

**Authors:** Walaa Mohamed Sayed Gomaa, Atef Mohamed Saleem, Tao Ran, Long Jin, Mohamed Samir, Emma J. McGeough, Kim Ominski, Lingyun Chen, Wenzhu Yang

**Affiliations:** ^1^Lethbridge Research and Development Centre, Agriculture and Agri-Food Canada, Lethbridge, AB, Canada; ^2^Department of Animal Nutrition and Clinical Nutrition, Assiut University, Assiut, Egypt; ^3^Department of Animal and Poultry Production, South Valley University, Qena, Egypt; ^4^College of Pastoral Science and Technology, University of Lanzhou, Lanzhou, China; ^5^Department of Zoonoses, Faculty of Veterinary Medicine, Zagazig University, Zagazig, Egypt; ^6^Department of Animal Science and National Centre for Livestock and the Environment, University of Manitoba, Winnipeg, MB, Canada; ^7^Department of Agricultural, Food and Nutritional Science, University of Alberta, Edmonton, AB, Canada

**Keywords:** red osier dogwood extract, DDGS, high grain diet, RUSITEC, fermentation pattern

## Abstract

The objective of this study was to evaluate the effect of dried distillers grains with solubles (DDGS) and red-osier dogwood (ROD) extract on *in vitro* fermentation characteristics, nutrient disappearance, and microbial profiles using the rumen simulation technique. The experiment was a completely randomized design with a 2 × 2 factorial arrangement of treatments and four replicates per treatment. A basal diet [10% barley silage, 87% dry-rolled barley grain, and 3% vitamin and mineral supplement, dry matter (DM) basis] and a DDGS diet (as per basal diet with 25% of wheat DDGS replacing an equal portion of barley grain) were supplemented with ROD extract at 0 and 1% (DM basis), respectively. The experimental period was 17 d, consisting 10 days of adaptation and 7 days of data and sample collection. The substitution of wheat DDGS for barley grain did not affect gas production; disappearances of DM, organic matter, and crude protein; total volatile fatty acid (VFA) production; and microbial protein production. However, replacing barley grain with wheat DDGS increased (*P* = 0.01) fermenter pH and molar proportion of branched-chain VFA, switched (*P* = 0.06) the fermentation pattern to higher acetate production due to increased (*P* = 0.01) disappearance of neutral detergent fiber (NDF), and decreased (*P* = 0.08) methane (CH_4_) production. In the basal barley diet, the ROD extract increased the acetate to propionate (A:P) ratio (*P* = 0.08) and reduced the disappearance of starch (*P* = 0.06) with no effect on any other variables. No effects of ROD in the DDGS diet were observed. The number of operational taxonomic unit (OTUs) and the Shannon diversity index of the microbial community had little variation among treatments. Taxonomic analysis revealed no effect of adding the ROD extract on the relative abundance of bacteria at the phylum level with either the basal diet or DDGS diet, while at the genus level, the microbial community was affected by the addition of both DDGS and the ROD extract. *Prevotella* and *Fibrobacter* were the most abundant genera in the basal diet; however, *Treponema* became the most abundant genus with the addition of the ROD extract. These results indicated that the substitution of wheat DDGS for barley grain may mitigate enteric CH_4_ emissions. The trend of reduced starch fermentability and increased NDF disappearance with the addition of ROD extract suggests a reduced risk of rumen acidosis and an improvement in the utilization of fiber for cattle-fed high-grain diet.

## Introduction

Red osier dogwood (ROD; *Cornus sericea*) is a native shrub plant that is present across North America and is abundant in low wetlands, pasturelands, and areas where crops and forages do not grow well (1). This plant is rich in bioactive compounds, with total phenolic concentrations of up to 22% of dry matter (DM), including anthocyanins, gallic acid, ellagic acid, quercetin, kaempferol, and cyanin (1). Gallic and ellagic acids have been shown to possess anti-inflammatory (2) and antioxidant (3) benefits (4). In recent years, ROD and its potential as a feed ingredient for livestock have been evaluated in *in vitro* and *in vivo* studies utilizing beef cattle and swine. Wei et al. (5) investigated the effect of substituting ROD for silage at 3% and 6–12% in a high-grain diet on *in vitro* rumen fermentation with varying media pH (5.8 vs. 6.5). They suggested that the inclusion of ROD at a media pH of 5.8 had greater effects on starch digestion than on fiber digestion. This is of particular interest as decreased ruminal starch digestion would alleviate rumen acidosis in animals that are fed high-grain diets, where the rumen pH is consistently below 5.8 and fibrolytic bacteria are compromised, potentially impacting the performance, as well as the health and welfare, of animals. Wei et al. (6) also reported that feeding ROD to beef heifers that are fed high-grain diets decreased the degradability of ruminal protein (i.e., increased the rumen bypass protein) and increased blood antioxidant capacity and the immune response. These authors suggested that the increase in rumen bypass protein was due to the protein-binding capacity of ROD phenols, thereby improving protein efficiency in cattle. However, these previous studies used raw ROD material, and to our knowledge, ROD extract and its effect on rumen fermentation have not been evaluated.

Dried distillers grains with solubles (DDGS) is the major by-product of ethanol production when corn or wheat grain is used as a substrate for ethanol production. The inclusion of DDGS in feedlot diets is a common practice, given its high protein and high digestible fiber concentrations, in addition to its competitive cost as a feed ingredient. However, diets with high inclusion levels of DDGS, especially wheat DDGS, which have a higher crude protein (CP) content than corn DDGS, often exceed CP requirements for cattle fed with barley grain-based feedlot finishing diets. Feeding CP in excess of cattle requirements is not nutritionally and environmentally desirable as it increases ruminal absorption of ammonia nitrogen (NH_3_-N) and, thus, increases nitrogen (N) excretion in a volatile form (7). We hypothesize that supplementation of ROD extract in high-grain diets containing DDGS may alter the ruminal fermentation pattern in a desirable manner and increase ruminal bypass protein by reducing the NH_3_-N concentration due to decreased proteolytic activity. Thus, the objective of this study was to investigate the effect of wheat DDGS, the supplementation of ROD extract, and their interaction on fermentation pattern, nutrient disappearance, and rumen microbial profile in an artificial rumen stimulation technique (RUSITEC).

## Materials and Methods

### Experimental Design, Diets, and ROD Extract

The experiment was a completely randomized design with a 2 × 2 factorial arrangement of four treatments with four replicates, which included two total mixed ration diets; a basal diet (87% dry-rolled barley grain, 10% barley silage, and 3% vitamin and mineral supplement; DM basis) and a wheat DDGS diet (62% barley grain, 25% wheat DDGS, 10% barley silage, and 3% supplement; DM basis), each supplemented with 0% and 1% ROD extract (DM basis), respectively. The ROD extract was supplied by Red Dog Enterprise Ltd. (Winnipeg, MB, Canada) and was prepared using hydrothermal treatment with an extraction temperature of 98°C. The ROD extract contained approximately 6.7% moisture, 21.8% total phenolic content (expressed as gallic acid equivalents), 1.67% gallic acid, 0.70% ellagic acid, 2.75% rutin, 0.32% quercetin malonyl glucoside, and 0.01% quercetin [DM basis; (8)]. The inclusion level of the ROD extract was based on a previous *in vitro* study carried out at our laboratory (9). The total mixed ration diets were ground through a 4-mm sieve (Arthur Thomas Co., Philadelphia, PA, USA) and weighed (10 g DM) into nylon bags (10 × 20 cm; pore size of 50 μm, Ankom Technology Corp., Macedon, NY, USA). The ROD extract was then added to nylon bags at the desired level and was manually mixed. The chemical composition of the experimental diets is reported in Table 1.

**Table 1:** Ingredient and chemical compositions of the experimental diets (% of DM unless otherwise stated).

**Item**	**Basal**	**DDGS**
**Ingredient**		
Barley silage^a^	10	10
Barley grain, ground^b^	87	62
Wheat DDGS^c^	0	25
Supplement^d^	3	3
**Chemical composition**		
DM, %	85.4	85.0
OM	94.3	92.3
CP	13.7	19.6
NDF	21.8	23.9
ADF	8.4	9.1
Starch	52.6	38.5
Ether extract	2.1	2.9

DDGS, dried distillers grains with solubles; DM, dry matter; OM, organic matter; CP, crude protein; NDF, neutral detergent fiber; ADF, acid detergent fiber.

a Composition (DM basis): 38.4% DM, 90.7% OM, 11.9% CP, 47.3% NDF, 24.8% ADF, and 20.5% starch.

b Composition (DM basis): 96.7% OM, 13.6% CP, 19.6% NDF, 6.8% ADF, and 58.1% starch.

c Composition (DM basis): 94.0% OM, 40.2% CP, 28.2% NDF, 9.8% ADF, 4.1% ether extract, and 1.7% starch.

d *Supplement consisted of (dietary DM) 0.92% ground barley, 0.83% canola meal, 1.04% calcium carbonate, 0.04% molasses, 0.09% salt, 0.02% feedlot premix (supplied per kilogram of dietary DM: 15 mg Cu, 65 mg Zn, 28 mg Mn, 0.7 mg I, 0.2 mg Co, 0.3 mg Se, 6,000 IU vitamin A, 600 IU vitamin D, and 47 IU vitamin E), 0.06% urea, 0.002% vitamin E (500,000 IU/kg), and 0.02% canola oil*.

### Experimental Apparatus

Two RUSITEC apparatus were used with each unit equipped with eight 920-ml fermenters as described by Czerkawski and Breckenridge (10). Each fermenter had an inlet for the infusion of the buffer and an outport for the collection of effluent. Fermenters were immersed in a water bath maintained at 39°C. The four treatments were randomly assigned to duplicate fermenters within each RUSITEC apparatus. The experimental period was 17 days, comprising 10 days for adaptation and 7 days for data and sample collection.

On day 1, a bag containing 20 g of wet solid rumen contents, a diet bag containing 700 ml ruminal fluid, and 200 ml of warmed McDougall's buffer (11) were added to each fermenter. Ruminal liquid and solid were added to inoculate the feed with liquid- and particle-associated microbes. The McDougall's buffer was modified to contain 0.82% NaH_2_PO_4_ + H_2_O (wt/vol), 0.63% NaHCO_3_ (wt/vol), and 0.03% (NH_4_)_2_SO_4_ (wt/vol). The McDougall's buffer was continuously infused into the fermenters at a dilution rate of 2.9%/h during the entire experimental period. Solid rumen content bags were removed after 24 h of incubation and replaced with a diet bag. Thereafter, each fermenter contained two diet bags with one bag being removed daily and a new bag inserted. Thus, the incubation time for each bag in the fermenter was 48 h. During bag exchange, anaerobic conditions were maintained by the infusion of CO_2_ into the fermenters. Fermented gases and accumulated effluents were collected into reusable 2 L gas-tight vinyl collection bags (Curity® Covidien Ltd., Mansfield, MA, USA) that were attached to each of the effluent vessels and Erlenmeyer flasks (2 L), and production was measured daily.

### Source of Inoculum

Solid and liquid ruminal contents were collected from different locations in the rumen (6 L per animal) at 2 h after morning feeding from three ruminal fistulated beef heifers (average, 698 ± 65.1 kg body weight). The heifers were fed a high-grain diet containing 15% barley silage, 82% barley grain, and 3% vitamin and mineral supplement (DM basis). The collected rumen contents were immediately filtered through four layers of cheesecloth, pooled, and pH recorded before dispensing into the fermenters.

### Fermentation Characteristics and N Fraction

Fermenter media pH was measured during bag exchange using a calibrated pH meter (Orion model 260A, Fisher Scientific, Toronto, ON, Canada). During the sampling period, the effluent was collected for the analysis of VFA, NH_3_-N, and soluble N. To determine VFA concentration, 5 ml of effluent was added to 1 ml of 25% metaphosphoric acid (HPO_3_) (wt/vol) in screw-capped vials and stored at −20°C until analysis. The VFA concentration was determined using a gas chromatograph (model 5890, Hewlett-Packard Labs, Palo Alto, CA, USA), with a capillary column (1-μm phase thickness, 30 m × 0.32 mm i.d., Zebron ZB-FFAP, Phenomenex, Torrance, CA, USA), a flame ionization detector, and trans-2-butenoic acid as an internal standard. To measure NH_3_-N concentration, 5 ml of effluent was added to 1 ml of 1% H_2_SO_4_ (wt/vol) in screw-capped vials and stored at −20°C until analysis using the modified Berthelot method (12). Daily VFA and NH_3_-N production were calculated by multiplying the concentration of the fermentation end product in the effluent by the daily production of effluent.

Large and small peptide N were determined by measuring soluble N in the effluent using tungstic acid (TA) and trichloroacetic acid (TCA), as described by Winter et al. (13) and Li et al. (14). Four milliliters of effluent was mixed with 1 ml of 10% (wt/vol) sodium tungstate and 1 ml of 1.07 N sulfuric acid to measured TA soluble N (TA-N). TCA soluble N (TCA-N) was measured by adding 1 ml of 50% (wt/vol) TCA solution to 4 ml of effluent. All tubes were kept at 5°C for 4 h and then centrifuged at 9,000 × g for 15 min. All supernatants were collected for the analysis of TA-N and TCA-N using flash combustion and a thermal conductivity detector (Model 1500; Carlo Erba Instruments, Milan, Italy). The large peptide N concentration was calculated as the difference between TCA-N and TA-N, while the small peptide N (including amino acid; AA) concentration was calculated by the difference between TA-N and NH_3_-N.

### Nutrient Disappearance and Gas Production

Dry matter disappearance (DMD) was determined from day 11 to day 15 of the sampling period. After 48 h of incubation, the bags were removed, gently squeezed, and washed under running cold tap water until the water was clear. The bags were oven-dried at 55°C for 48 h and weighed to determine the DMD. The residue in the bags was removed, pooled by using the fermenter, and ground through a 1-mm sieve (standard model 4, Arthur Thomas Co., Philadelphia, PA, USA) for chemical analysis. The experimental diets and the residue from the bags were analyzed for DM (method no. 930.15) and ash content (method no. 942.05) according to the AOAC (15). Neutral detergent fiber (NDF) concentration was determined according to the method described by Van Soest et al. (16) using heat-stable amylase and sodium sulfite and expressed inclusivity of residual ash. The concentration of acid detergent fiber (ADF) was determined according to method no. 973.18 (15). A combustion analyzer (NA 2100, Carlo Erba Instruments, Milan, Italy) was used for total *N* analysis (method no. 990.03; 15), and the CP was calculated as *N* × 6.25. The starch was determined by enzymatic hydrolysis of α-linked glucose polymers as reported previously (17). The fat content was determined using ether extraction (Extraction Unit E-816, Büchi Labortechnik AG, Flawil, Switzerland; 15; method no. 920.39). Nutrient disappearance was calculated as the difference between nutrient concentration in the substrate before and after incubation.

Daily gas production (GP) was measured during bag exchange in the morning using a wet-type gas meter (Alexander-Wright, London, UK). From day 11 to day 15, a gas sample (20 ml) was collected from each bag using a 26-gauge needle (Becton Dickinson, Franklin Lakes, NJ, USA) which is injected into evacuated 6.8-ml Exetainer vials (Labco Ltd., High Wycombe, Bucks, UK). The methane (CH_4_) concentration in the gas samples was determined, as described by Avila-Stagno et al. (18), using a Varian CP-4900 mICRO Gas Chromatograph equipped with a GS-CarbonPLOT 30 m × 0.32 mm × 3 μm column and a thermal conductivity detector (Agilent Technologies Canada Inc., Mississauga, ON, Canada).

### Microbial Protein Synthesis

Feed particle-associated (FPA) bacteria, feed particle-bound (FPB) bacteria, and effluent liquid-associated bacteria (LAB) were evaluated to determine microbial protein synthesis. The bacteria were labeled with ^15^N, and on day 10 of the experiment, samples were collected from the effluent and substrate residue to determine the analysis of background ^15^N. On day 11, (^15^NH_4_)_2_SO_4_ (Sigma Chemical Co., St. Louis, MO, USA; minimum ^15^N enrichment 1 g/L) was added to the McDougall's buffer instead of (NH_4_)_2_SO_4_ until the end of the sampling period, and sodium azide was added to effluent flasks to stop microbial activity (3 ml/flask per day; 0.1% wt/vol final concentration).

On days 16 and 17, 35 ml of effluent were sampled for LAB determination. The effluents were centrifuged at 20,000 × g at 4°C for 30 min, and the resulting pellets were washed using deionized water and centrifuged three times (20,000 × g, 30 min, 4°C). The pellets were suspended in distilled water and freeze-dried until N and ^15^N analysis. FPA bacteria and FPB bacteria were measured by removing the bags from the fermenters and gently squeezing to expel excess liquids. Thereafter, bags were weighed and placed individually in plastic bags with 20 ml of phosphate buffer and were processed two times in a Stomacher 400 Circulator laboratory blender (Seward Medical Ltd., London, UK) for 1 min each time. The bags were then manually squeezed and washed two times with phosphate buffer. The squeezed and rinsed liquid (FPA fraction) was centrifuged at 500 × g at 4°C for 10 min to remove large feed particles, and the supernatant was centrifuged (20,000 × g, 30 min, 4°C) to isolate a bacterial pellet, which was washed three times as previously described. The pellets were suspended in distilled water and freeze-dried until N and ^15^N analysis. The washed feed residues (FPB fraction) were oven-dried at 55°C for 48 h for DMD, and they were also ball ground (MM400; Retsch Inc., Newtown, PA, USA) for N and ^15^N analysis. The flash combustion method (Model 1500; Carlo Erba Instruments, Milan, Italy) was used for total N determination. The ^15^N enrichment was measured by ^15^N continuous flow measurement using a combustion analyzer interfaced with a mass spectrometer (VG Isotech, Middlewich, UK).

### Microbial Community

High-throughput sequencing techniques were used to evaluate FPA microbial communities. Thirty milligrams of the FPA samples were obtained for total DNA extraction and were analyzed with the QIAamp Fast DNA Stool Mini Kits (Qiagen, Hilden, Germany) as per the instructions provided by the manufacturer. Before the DNA extraction, the freeze-dried rumen samples were bead beat for 2 min at 50 Hz to break down any fiber particles that may block the DNA-binding column during DNA extraction. The concentration and purity of the DNA were checked using a NanoDrop spectrophotometer (Thermo Fisher Scientific, Waltham, MA, USA), and the DNA samples were stored at −20°C until sequencing analysis. The PCR technique and DNA sequencing were completed following the method defined by Walters et al. (19). Amplification of the V4 region of the bacterial 16S rRNA gene was conducted using modified 515-F and 806-R primers. About 99% OTUs of Greengenes 13_8 from the 515F/806R region of the sequence database were used, and no difference in methanogens was found among treatments. A two-step PCR was followed to produce the 165 rRNA gene amplicons, which were then exposed to Illumina paired-end library preparation and cluster generation. The Illumina MiSeq instrument was used for sequencing (Illumina, Inc., San Diego, CA, USA). The R package DADA2 (Version 1.4) denoising method and QIIME2 were used for treating the raw data for gene sequencing according to Bolyen et al. (20). Primer sequences were removed, forward and reverse reads were truncated at 225 bp, and the read quality was checked using QIIME2. The number of OTUs (richness) and the Shannon index (diversity) were also calculated. Non-metric multidimensional scaling (NMDS) was carried out using the R packages vegan (Version 2.4.4) and phyloseq (Version 1.20.0) according to the Bray–Curtis similarity distances. Ruminal bacterial fold change at the genus level with a 5% threshold was considered using the R package Deseq2 (21).

### Statistical Analysis

The data were analyzed using the MIXED procedure of SAS (SAS Inst. Inc., Cary, NC, USA) as repeated measures according to a 2 × 2 factorial arrangement of treatments. The model included wheat DDGS, ROD extract, and their interaction as fixed effects, with the fermenter and the RUSICTEC apparatus as random effects. Sampling days were used as repeated measures for GP profiles, fermenter pH, VFA production and profiles, NH_3_-N production, and DMD parameters. Several covariance structures, such as the compound symmetry, heterogeneous compound symmetry, autoregressive, heterogeneous autoregressive, and unstructured, were carried out for repeated measures, and a low Akaike's information criterion value was chosen. Results were declared as least square means, and the significance was reported at *P* ≤ 0.05 and a trend was reported at 0.05 < *P* ≤ 0.10, unless otherwise stated.

## Results

### Fermentation Characteristics and N Fraction

Fermenter pH was higher (*P* = 0.01) with DDGS than the basal diet, with the supplementation of ROD extract having no effect (Table 2). Total VFA production and the molar proportion of acetate, propionate, and butyrate were not affected by the diets or supplementation with the ROD extract, except for a tendency for the butyrate to be higher (*P* = 0.08) with ROD extract supplementation; no interaction between the ROD extract and the diets was observed. Basal diet had a lower (*P* = 0.01) molar proportion of branched-chain VFA (BCVFA) and valerate but a higher (*P* = 0.01) proportion of caproate compared to the DDGS diet, with the supplementation of the ROD extract reducing (*P* = 0.01) caproate in both diets. The production of NH_3_-N was higher (*P* = 0.01) with DDGS compared to the basal diet, with no observed effect of the ROD extract. The large peptide N was higher (*P* = 0.01) with DDGS compared to the basal diet, with the opposite outcome (*P* = 0.02) observed for the small peptide N fraction. No interactions were observed in the N fraction.

**Table 2:** Effect of wheat dried distillers grains with solubles (DDGS) and the red osier dogwood (ROD) extract on fermentation and nitrogen fraction in RUSITEC.

**Item**	**Basal**^****a****^		**DDGS**^****a****^			***P*****-value**		
	**–ROD**	**+ROD**	**–ROD**	**+ROD**	**SEM**	**DDGS**	**ROD**	**Inter**
pH	5.74	5.75	5.90	5.90	0.02	0.01	0.95	0.99
Total VFA, m*M*/d	45.6	44.0	44.7	43.5	1.42	0.63	0.35	0.89
**VFA, mol/100 mol**								
Acetate (A)	27.8	28.9	29.0	28.9	0.33	0.10	0.19	0.10
Propionate (P)	41.2	39.9	40.1	39.6	0.58	0.25	0.49	0.15
Butyrate	20.1	21.0	19.8	20.5	0.42	0.39	0.08	0.72
BCVFA	2.1	2.2	2.3	2.3	0.04	0.01	0.42	0.42
Valerate	5.8	5.2	6.8	6.9	0.30	0.01	0.50	0.28
Caproate	3.3	2.6	2.0	1.8	0.14	0.01	0.01	0.10
A:P	0.67	0.72	0.72	0.73	0.02	0.06	0.15	0.08
NH_3_ N production, mg/d	54.7	54.6	91.3	92.0	2.10	0.01	0.87	0.88
**N fraction, mg/100 ml**								
Large peptide	22.2	22.9	15.1	16.5	1.06	0.01	0.33	0.75
Small peptide	13.2	11.8	15.4	14.5	0.97	0.02	0.25	0.83
NH_3_-N	8.2	8.0	13.5	13.4	0.33	0.01	0.79	0.94

SEM, standard error of the mean; Inter, interaction between DDGS and ROD; VFA, volatile fatty acid; BCVFA, branched-chain VFA (isobutyrate + isovalerate); DM, dry matter; N, nitrogen; NH_3_-N, ammonia, N.

a *The diet consisted of barley silage, barley concentrate, and wheat DDGS in the ratios of 10:90:0 and 10:65:25, respectively, for the basal and DDGS diets (DM basis); the ROD extract was added with 0 (–ROD) or 1% dietary DM (+ROD)*.

### Nutrient Disappearance and Gas Production

There was no significant effect of DDGS and ROD extract on nutrient disappearance of DM, organic matter (OM), ADF, and CP (Table 3). In contrast, the NDF disappearance was increased (*P* = 0.01) by partially replacing barley grain with wheat DDGS. No interactions were observed except for a tendency (*P* = 0.06) of starch disappearance. The NDF disappearance was (*P* = 0.10) higher but starch disappearance was lower (*P* = 0.06) when supplementing ROD extract in the basal diet, but with no effect in the DDGS diet. No effects of DDGS or ROD extract supplementation on GP and CH_4_ production (mg/d) were observed. There was a tendency for DDGS to have higher CH_4_, expressed as percentage of gas (*P* = 0.05) and mg/g digested basis (*P* = 0.08), compared to the basal diet. No interactions between the basal diet and ROD were observed.

**Table 3:** Effect of wheat DDGS and the ROD extract on nutrient disappearance and gas production (GP) in RUSITEC.

**Item**	**Basal**^****a****^		**DDGS**^****a****^			***P*****-value**		
	**–ROD**	**+ROD**	**–ROD**	**+ROD**	**SEM**	**DDGS**	**ROD**	**Inter**
**Nutrient disappearance, %**								
DM	76.3	77.5	77.3	77.5	0.69	0.43	0.35	0.45
OM	77.5	78.6	77.8	77.9	0.71	0.83	0.40	0.52
NDF	24.5	27.6	40.8	40.2	1.07	0.01	0.26	0.10
ADF	22.5	25.3	26.5	26.2	1.46	0.76	0.40	0.31
Starch	90.8	88.9	88.4	87.2	0.81	0.19	0.21	0.06
CP	82.5	83.1	82.5	82.1	1.06	0.64	0.97	0.65
**Gas production**								
GP, L/d	1.76	1.75	1.70	1.74	0.07	0.55	0.87	0.73
GP, ml/g DM digested	230.6	223.1	218.8	221.4	7.61	0.39	0.75	0.52
CH_4_, % of gas	1.95	2.11	1.75	1.79	0.12	0.05	0.42	0.61
CH_4_, mg/d	34.4	37.4	30.1	31.1	3.07	0.11	0.52	0.73
CH_4_, mg/g DM digested	2.93	3.09	2.52	2.59	0.25	0.08	0.65	0.83

SEM, standard error of the mean; Inter, interaction between DDGS and ROD; DM, dry matter; OM, organic matter; CP, crude protein; NDF, neutral detergent fiber; ADF, acid detergent fiber; CH_4_, methane; GP, gas production.

a *The diet consisted of barley silage, barley concentrate, and wheat DDGS in the ratios of 10:90:0 and 10:65:25, respectively, for the basal and DDGS diets (DM basis); ROD extract was added with 0 (–ROD) or 1% dietary DM (+ROD)*.

### Microbial Protein Synthesis

The production of total microbial N and LAB was not affected by the inclusion of DDGS, whereas FPA production decreased (*P* = 0.06) and the FPB production increased (*P* = 0.01) with the addition of DDGS compared with the addition of basal diets (Table 4). The production of microbial N was not affected by supplemental ROD extract, and the efficiency of microbial N synthesis did not differ among treatments. No interactions between DDGS and ROD extract diets were observed.

**Table 4:** Effect of wheat DDGS and the ROD extract on microbial N synthesis in RUSITEC.

**Item**	**Basal**^****a****^		**DDGS**^****a****^			***P*** **value**		
	**–ROD**	**+ROD**	**–ROD**	**+ROD**	**SEM**	**DDGS**	**ROD**	**Inter**
**Microbial N, mg/d**								
Total	81.4	80.2	84.4	86.6	5.74	0.38	0.92	0.72
LAB	66.8	64.9	71.0	71.2	5.20	0.36	0.87	0.83
FPA	12.1	12.7	9.7	11.0	1.10	0.06	0.38	0.72
FPB	2.3	2.8	4.1	4.3	0.16	0.01	0.12	0.36
EMPS, mg N/g OM digested	10.2	10.0	10.8	11.2	0.70	0.18	0.82	0.61

SEM, standard error of the mean; Inter, interaction between DDGS and ROD; N, nitrogen; LAB, liquid-associated bacteria; FPA, feed particle-associated bacteria; FPB, feed particle-bound bacteria; EMPS, efficiency of bacterial N synthesis; OM, organic matter.

a *The diet consisted of barley silage, barley concentrate, and wheat DDGS in the ratios of 10:90:0 and 10:65:25, respectively, for the basal and DDGS diets (DM basis); ROD extract was added with 0 (–ROD) or 1% dietary DM (+ROD)*.

### Microbial Community

**Figure 1:**
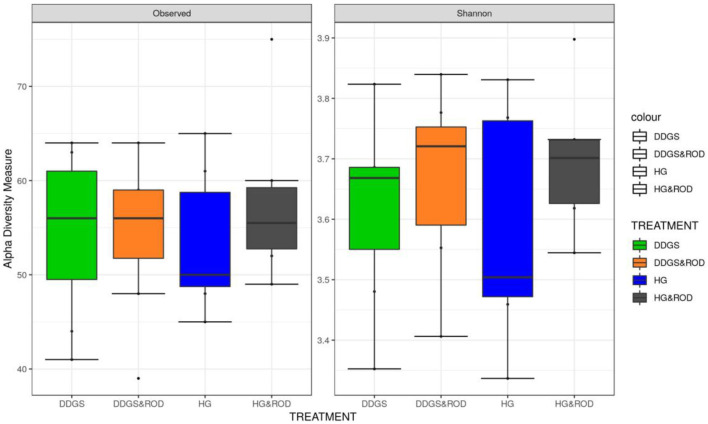
Box plots of the number of operational taxonomic units (OTUs) and the Shannon diversity index for feed particle-associated (FPA) samples by treatment. Treatments were as follows: wheat dried distillers grains with solubles (DDGS) diet without adding red osier dogwood (ROD) extract; DDGS&ROD (wheat DDGS diet with ROD extract); HG (basal diet without adding ROD extract); and HG&ROD (basal diet with ROD extract).

**Figure 2:**
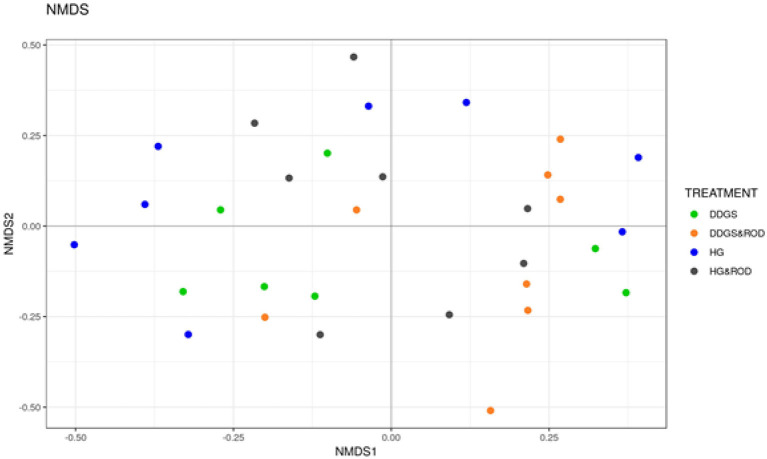
Non-metric multidimensional scaling (NMDS) plots of the Bray–Curtis dissimilarities for FPA samples by treatment. Treatments were as follows: DDGS diet without adding the ROD extract; DDGS&ROD (wheat DDGS diet with ROD extract); HG (basal diet without adding ROD extract); and HG&ROD (basal diet with ROD extract).

The number of OTUs (Figure 1, Observed) and the Shannon diversity index (Figure 1, Shannon) showed that the microbial community had little variation among treatments (variance in the mean of Shannon diversity index = 0.002, variance in the median of Shannon diversity index = 0.009). However, the addition of ROD extract to the DDGS diet resulted in the highest diversity in bacterial species (Median Shannon index = 3.7), while the lowest diversity (highest homogeneity) was observed with the basal diet (Median Shannon = 3.5). Similarly, the NMDS plot depicted in Figure 2 showed no separation or absence of specific clustering of FPA microbial communities among treatments, indicating a high similarity between samples taken from the various diets in terms of the identified bacterial genera.

**Figure 3:**
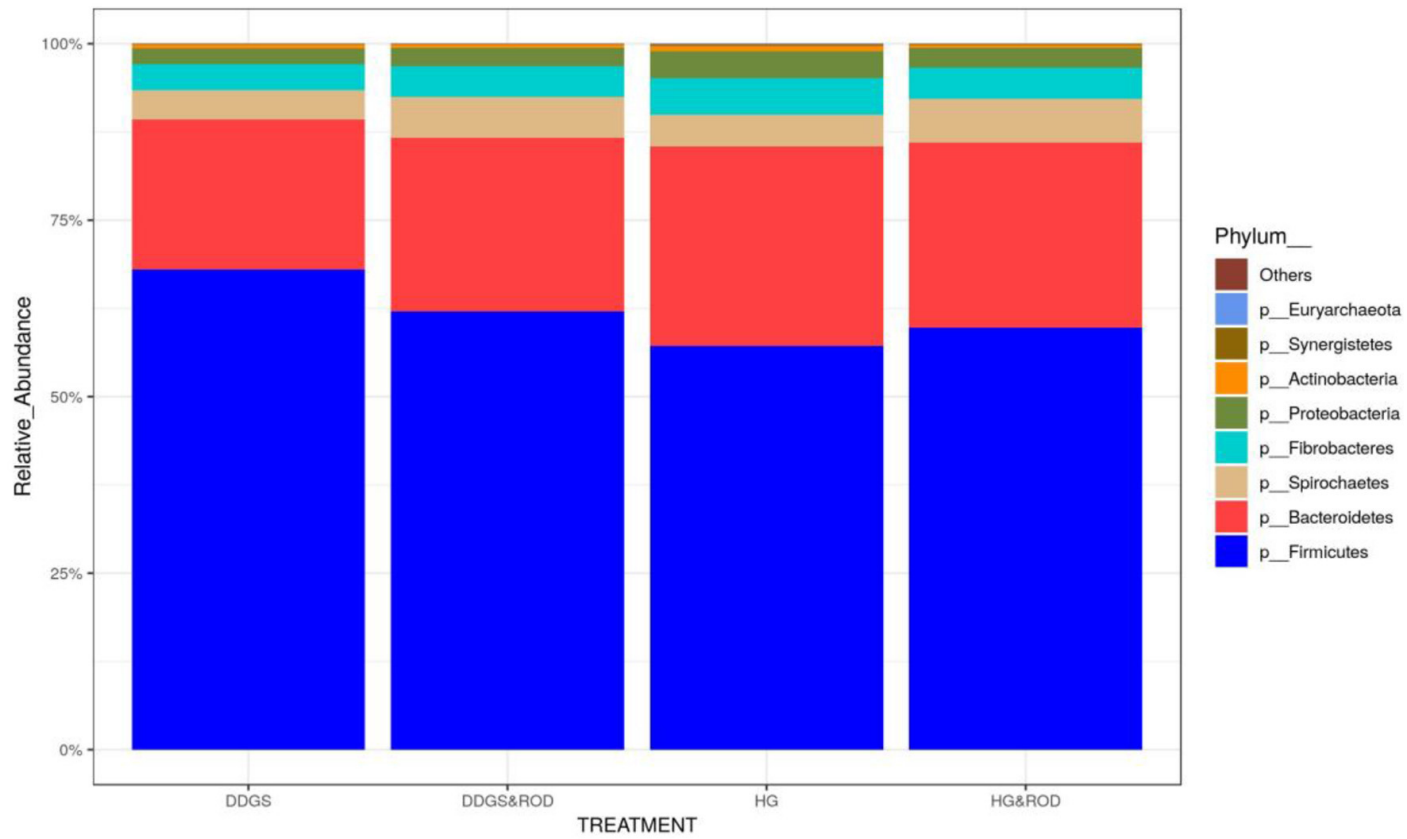
Effect of different treatments on the relative abundance (RA) of the ruminal microbial community at the phylum level. Treatments were as follows: wheat DDGS diet without adding the ROD extract; DDGS&ROD (wheat DDGS diet with ROD extract); HG (basal diet without adding ROD extract); and HG&ROD (basal diet with ROD extract).

**Figure 4:**
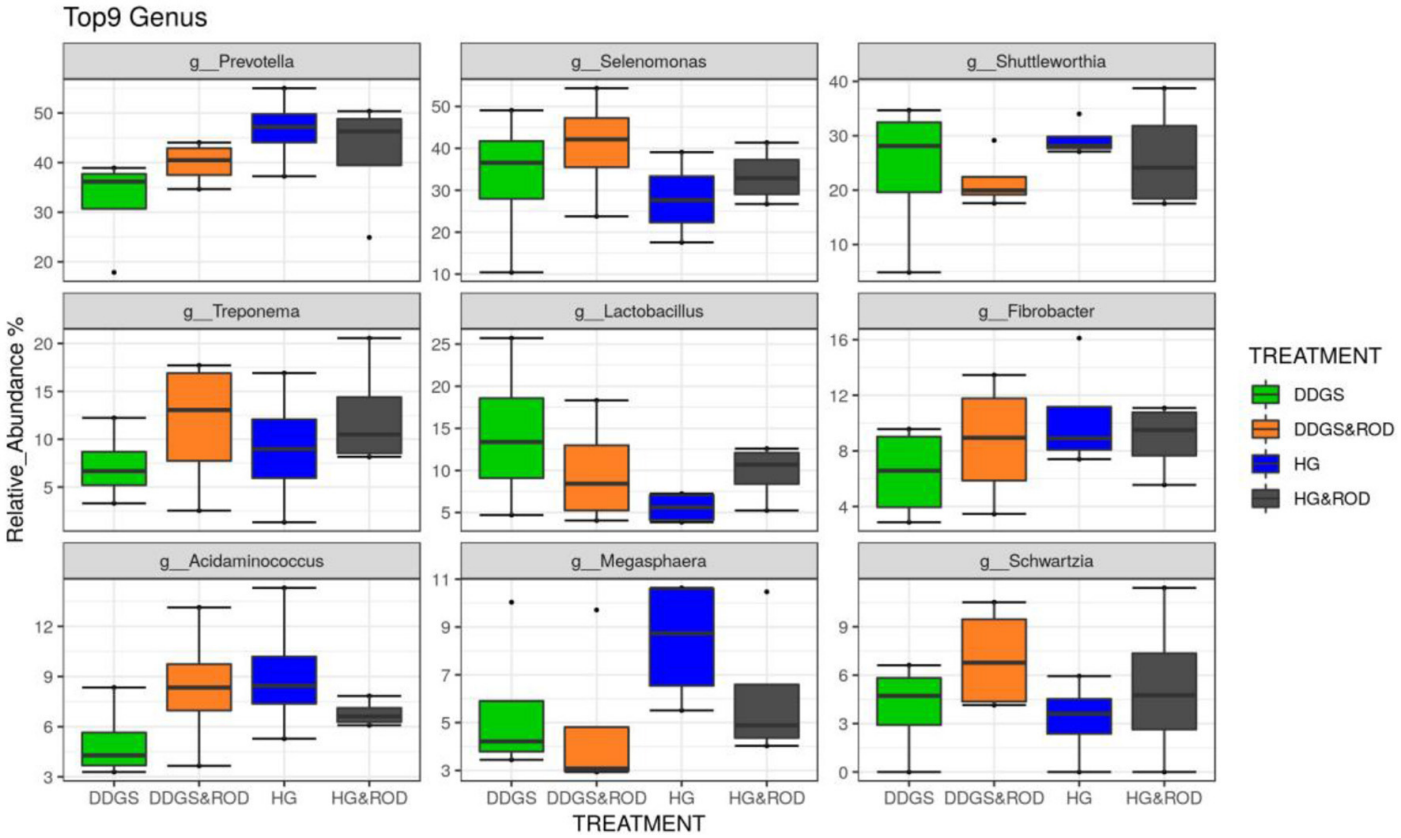
Effect of different treatments on the RA of the ruminal microbial community at the genus level. Treatments were as follows: wheat DDGS diet without adding the ROD extract; DDGS&ROD (wheat DDGS diet with ROD extract); HG (basal diet without adding ROD extract); and HG&ROD (basal diet with ROD extract).

Taxonomic analysis, as shown in Figure 3, showed that the phylum Firmicutes exhibited the highest abundance (abundance = 62.7) among all other phyla irrespective of the diet, followed by the phylum Bacteroidetes (abundance = 24.6). Other phyla existed with very low abundance (range of 3.9–0), indicating that the addition of the ROD extract had no effect on the relative abundance of the bacteria at the phylum level. At the genus level, *Prevotella* (median = 26.4) and *Fibrobacter* (median = 5) were the most abundant genera in the basal diet; however, *Treponema* became the most abundant genus (Figure 4) with the addition of the ROD extract. The dominant genera associated with the DDGS diet were *Acidaminococcus* (median = 4.9), *Megasphaera* (median = 4.8), *Shuttleworthia* (median = 18.2), and *Lactobacillus* (median = 6.8). However, *Selenomonas* (median = 27.9), and *Schwartzia* (median = 4.2) were the dominant genera when the ROD extract was added to the DDGS diet.

**Figure 5:**
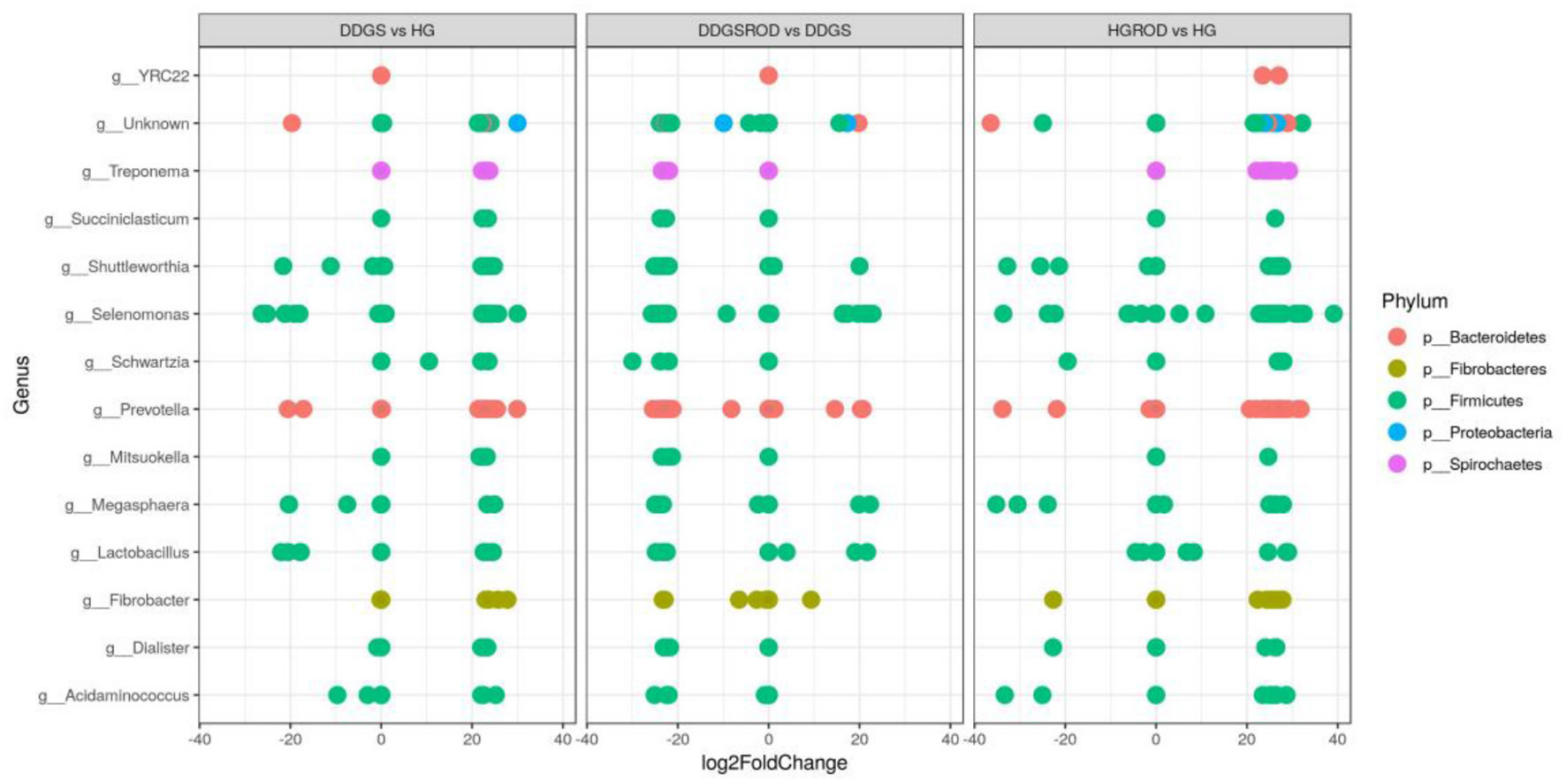
Relative changes (log2 fold; *P* < 0.05) of ruminal bacteria by DDGS vs. HG, DDGS&ROD vs. DDGS, and HG&ROD vs. HG at the genus level. DDGSDDGS diet without adding the ROD extract; DDGS&ROD (wheat DDGS diet with ROD extract); HG (basal diet without adding the ROD extract); HG&ROD (basal diet with the ROD extract).

The log2 fold change analysis indicated that the majority (97.3%) of the microbial community that differed (*P* = 0.05) between the basal and DDGS diets were upregulated while only five (2.6%) were downregulated (Figure 5). The upregulation was most prevalent in *Prevotella* (phylum Bacteroidetes), while *Selenomonas* (phylum Firmicutes) showed the highest downregulation. The addition of ROD extract resulted in changes in the microbial community in both the basal or DDGS diets, although the impact was greatest in the DDGS diet. Supplementation of the ROD extract in the DDGS diet increased all genera that belonged to the phylum Firmicutes (especially genera *Selenomonas*) but decreased the genus *Prevotella*, which belongs to phylum Bacteroidia. Adding ROD extract to the basal diet resulted in the upregulation of *Fibrobacter* (phylum Fibrobacteres), whereas *Shuttleworthia* (phylum Firmicutes) was the most downregulated genus.

## Discussion

### Effects of Wheat DDGS

The wheat DDGS diet exhibited higher rumen pH than the basal diet, which is likely reflective of its lower starch content. Increasing dietary starch concentration is well-established to have a negative effect on rumen pH (22). Relative change between pH and VFA concentration appeared to be similar with a 2% increase in pH and a 2% decrease in VFA concentration, indicating that the change in pH was primarily associated with decreased total VFA production. The increased NDF disappearance of the DDGS diets could be explained by a large fraction of digestible NDF in wheat DDGS (14) as well as the higher fermenter pH observed in the DDGS diets. Rumen cellulolytic bacteria are sensitive to rumen pH and adversely impacted at pH <6.0 (23), with pH of 5.8, 5.6, and 5.2 associated with mild, moderate, and severe subacute rumen acidosis, respectively (24).

In the rumen, the end products of feed substrates include VFA, gas (CH_4_), and microbial biomass. The production of VFAs plays an important role in supplying the animal with the energy needed for vital physiological processes (25). The main factors affecting total VFA production in the rumen are diet composition and the digestibility and fermentability of feedstuffs (26). The lack of difference between basal and DDGS diets in total VFA production and OM disappearance suggested that the two diets had similar nutrient fermentability. Rumen VFA profiles and fermentation patterns (i.e., acetate to propionate [A:P] ratio) in cattle offered diets containing wheat DDGS vary, likely depending on the composition and the proportion of wheat DDGS in the diets (27–29). A higher A:P ratio with DDGS than the basal diet was also observed by Beliveau and McKinnon (29), who reported that the substitution of wheat DDGS from 0%, 7%, 14–21% of dietary DM for barley grain increased the A:P ratio due to increased molar proportion of acetate and decreased propionate proportion. In the present study, however, the trend of increased acetate concentration and the A:P ratio in the DDGS compared to those of the basal diet was lower than expected, given the higher rate of NDF disappearance. Additionally, increased BCVFA apparent with the DDGS diet likely resulted from increased ruminal protein degradation associated with a high dietary CP content. The formation of BCVFA occurs during protein degradation as a result of branched-chain AA deamination (30). The higher valerate concentration with DDGS diets is in agreement with another study on RUSITEC using wheat DDGS with a similar treatment design but with the addition of a plant essential oil [cinnamaldehyde; (28)]. This may be explained by the higher soluble fraction (e.g., potentially high in sugars) in wheat DDGS than the parent material (31), leading to an increase in the concentration of valerate in the rumen (32).

The similar disappearance of DM and OM is consistent with the absence of differences in the disappearance of starch and CP between basal and DDGS diets. Although the NDF disappearance of the DDGS diet was higher than that of the basal diet, its quantitative contribution to the DM disappearance was smaller compared with starch and CP. The effect of adding wheat DDGS in barley grain-based diet on rumen DM digestibility is inconsistent in the literature (14, 28, 33). The discrepancy among studies may be due to the variation in the chemical composition of wheat DDGS, experimental conditions (14), and starch digestibility in grain vs. NDF digestibility of DDGS (33). Nevertheless, the ruminal NDF digestibility of wheat DDGS has been reported to be higher than that of feed grain or silage (33, 34) owing to the high hemicellulose in DDGS, which is more digestible than cellulose. The CP disappearance was high (>82%) in the current study and was not affected by substituting wheat DDGS in the barley grain diet, suggesting that the NDF insoluble N was low. Furthermore, the higher small peptide and NH_3_-N concentrations with the DDGS diet reflected the higher CP content and is in agreement with a previous study evaluating the effect of partly replacing barley grain with wheat DDGS in a high-grain diet (28).

The production of CH_4_ has a negative impact on the energy efficiency in ruminants and represents a loss of dietary energy ranging from 2 to 12% of gross energy intake (35). Supplementation of diets with lipids that are not protected from ruminal digestion is one strategy recognized to lower enteric CH_4_ emissions (36), and the reduction in CH_4_ by adding wheat DDGS in the place of barley grain is hypothesized due to a higher fat contribution from the DDGS. This has been reported by McGinn et al. (37), who substituted 35% of corn DDGS for barley grain in the diet of growing beef cattle, leading to a 16% (units) reduction in enteric CH_4_ production, which was attributed to the increased fat content (5.1%) of the diet. Some poly-unsaturated fatty acids in corn DDGS oil (especially linolenic acid) have been reported to specifically inhibit methanogenesis (38). Dugan et al. (39) reported that increasing wheat DDGS inclusion in beef cattle diets increased the percentage of linoleic acid from 51 to 56% of total fatty acids.

Adding wheat DDGS to barley-based diets has been reported to increase microbial protein production associated with improved OM digestibility (14, 28). The failure to increase microbial protein production with the DDGS vs. basal diets could be explained by the similar, but high, OM digestibility between basal and DDGS diets, thus denoting that energy is not be a limiting factor in microbial protein synthesis in the present study.

Although the ruminal microbial ecosystem can be affected by numerous factors, such as animal species, sex, stress, and environment (40), the most important factor is the type of diet (41). Henderson et al. (42) indicated that the diversity of the ruminal microbial community could be enhanced by diet complexity. In the current study, the diversity of the microbial community was not different among diets, which is in agreement with the study by Li et al. (28), who reported similar copy numbers of 16S rDNA of *Fibrobacter* and *Prevotella* between basal and wheat DDGS diets. However, when adding a plant essential oil (cinnamaldehyde) to either diet, copy numbers of *Fibrobacter* and *Prevotella* decreased (28). *Prevotella* exhibited upregulation while *Selenomonas* exhibited downregulation with the DDGS diet, indicating that the copy numbers of *Prevotella* may have responded to the higher CP content of the wheat DDGS. This result is in agreement with that from the study of Callaway et al. (43), who indicated that the *Prevotella* population could be enhanced by high CP concentrations. While *Selenomonas* is responsible for the fermentation of monosaccharides and disaccharides, as well as the production of propionate, the decrease in *Selenomonas* population explains the similarity in the propionate concentration.

### Effects of ROD Extract

It is well-established that supplementing feed additives can alter individual VFA profiles and fermentation patterns (5, 9). For example, it is well-known that feeding monensin to ruminants increases propionate production and decreases the A:P ratio, thus improving energy efficiency (5). The trend of increased acetate concentration and the A:P ratio due to the addition of the ROD extract to the basal diet in the present study is in agreement with the previous batch culture study (5). These findings suggested that starch disappearance has decreased and the NDF disappearance has increased because of adding ROD, which did occur in the current study. The reduction in starch digestibility because of adding the ROD extract was consistent with the changes of the microbial community at the genus level. *Treponema* was observed to be the most abundant genus, replacing the genera *Prevotella* and *Fibrobacter*. *Prevotella* at the genus level is usually dominant in the rumen microbial community associated with high-grain diets (44), as they are able to utilize various nutrients such as starch, proteins, and non-cellulosic polysaccharides (45).

Contrary to the previous batch culture study, where DMD and VFA production decreased by adding ROD to a high-grain diet (5), the disappearances of DM and OM and total VFA production were not affected by adding the ROD extract in the present study. The discrepancy between the two studies may be related to variations in the concentration of phenolic compounds between raw ROD and ROD extract, diet composition, or the *in vitro* technique used. Singh et al. (46) reported that the response of total VFA production to plant polyphenolic extracts varied with the original plant species and the level of inclusion of the extract. In the batch culture study conducted by Wei et al. (5), the fermentation period was shorter (i.e., 24 h), whereas, in the current study using RUSITEC, the data and samples were collected for 7 days followed by 10 days of microbial adaptation, thus indicating that the rumen microorganisms may have adapted to the treatments. Furthermore, the different levels of inclusion of phenolics used and their profiles may also lead to the variation between studies. In the previous study (5), the total amount of phenol used was 31, 62, or 124 mg/L, compared to 33 mg/L of fermentation media in the present study. The phenol composition appeared to be different between raw ROD and ROD extract; the phenolic compounds were identified as having a higher number of active compounds (gallic acid, methyl gallate, catechin, epicatechin, rutin, ellagic acid, and quercetin) in raw ROD compared to the ROD extract (gallic acid, ellagic acid, rutin, quercetin malonyl glucoside, and quercetin). These results suggest that, similar to raw ROD, adding ROD extract to a high-grain diet can impact rumen fermentation and microbial activity, which may vary with the phenolic compound profile. In the present study, the addition of the ROD extract led to a change in the dominant genera bacteria in both the basal and DDGS diets, indicating selective promotion or suppression of specific bacteria performed by the ROD extract.

Phenolics have both antioxidant and antimicrobial properties (2, 4); however, studies regarding the effect of these compounds on rumen fermentation and microbial activity are scarce (47). Furthermore, no other studies have assessed the effect of the ROD extract on the ruminal microbial community. No effect of adding the ROD extract on the relative abundance of bacteria at the phylum level is consistent with the absence of differences in GP, nutrient disappearance, and microbial protein synthesis. However, at the genus level, changes in the microbial community were observed, and it revealed altered microbial activity with the addition of the ROD extract. Such activity may not be necessary to significantly increase rumen nutrient degradation under present experimental conditions, especially with highly fermentable carbohydrates that are present in high-grain diets. Furthermore, the rumen is recognized to be an anaerobic environment; however, some oxygen may enter through feeds causing oxidative stress. Therefore, the ROD extract, which has antioxidants that reduce ruminal oxidative stress, may improve rumen function. However, the absence of effects of feeding the ROD extract on ruminal pH and VFA profiles suggested that low oxidant stress occurred in the rumen under the RUSITEC conditions of the present study.

### Interaction of DDGS Inclusion and ROD Extract

A number of interaction trends were observed between dietary DDGS and ROD extract supplementation, which were consistent with the study of Li et al. (28), who reported that *in vitro* rumen NDF digestibility and protozoan counts responded differently to the supplementation of plant essential oil (cinnamaldehyde) when barley grain was partly replaced with wheat DDGS in a high-grain diet. Both plant essential oil and phenols, plant-derived secondary active components, have demonstrated antimicrobial and antioxidant activity in livestock animals (28, 46, 47). The interaction of DDGS with the ROD extract and its effect on the A:P ratio and the disappearances of NDF and starch were expected. A previous study observed that increasing the substitution of ROD for barley silage in a high-grain diet decreased the *in vitro* propionate concentration and DMD but linearly increased the A:P ratio, suggesting decreased that the starch digestibility (5). In the present study, the basal diet contained higher starch and thus would be more likely to be affected by adding ROD extract. Concurring with the previous study, adding ROD extract increased the NDF disappearance of the basal diet with no effect in the DDGS diet, while reduced starch disappearance was more pronounced with the basal diet compared with the DDGS diet. Decreased rumen starch digestion with the addition of the ROD extract may potentially help alleviate rumen acidosis in beef cattle that are fed high-grain rations. Moreover, Li et al. (28) suggested that wheat DDGS may contain some exogenous components, such as yeast cells, to stimulate rumen microbial activity, thus possibly offsetting the ROD extract activity in diets containing DDGS, which would explain the absence of the effect of DDGS when the ROD extract was added.

The lack of interaction between DDGS and the ROD extract supplementation on protein degradability was somewhat unexpected, as Wei et al. (6) reported that beef heifers that were fed ROD with high phenol concentrations had lower rumen NH_3_-N concentration. The authors suggested that the supplementation of ROD to beef cattle that are fed high-grain diets may improve microbial protein synthesis or induce an increase in rumen bypass protein due to the protein-binding capacity of ROD phenols. Therefore, differences in the CP content between the basal and DDGS diets (basal vs. DDGS; 13.9 vs. 19.6% CP) were expected to result in differences in NH_3_-N concentration, microbial protein synthesis, or CP degradability. Gomaa et al. (48) reported that the *in situ* rumen degradation rate linearly decreased for silage protein, but it was not changed for grain protein when heifers were fed diets containing ROD. Wei et al. (5) suggested that phenols in ROD have a greater protein-binding capacity with soluble protein than with insoluble protein. The percentage of soluble protein was 3.5 and 6.4% of DM, respectively, for wheat grain and wheat DDGS (33).

## Conclusions

Replacing barley grain with wheat DDGS in a high-grain diet increased fermenter pH and molar proportion of BCVFA and switched the fermentation pattern to higher acetate production due to increased disappearance of NDF. The DMD and microbial protein production were not affected, whereas CH_4_ production tended to decrease with the inclusion of wheat DDGS. In conclusion, the substitution of wheat DDGS for barley grain at 25% of DM in high-grain diets potentially mitigates CH_4_ emissions. The supplementation of ROD extract at the rate of 1% of diet DM did not affect the GP; total VFA production; the disappearance of DM, OM, and CP; and microbial protein synthesis but increased the NDF disappearance, decreased starch disappearance, and increased the A:P ratio of the basal diet without DDGS. The decreased starch fermentability and the increased NDF disappearance as a result of adding the ROD extract suggest that the ROD extract may be used to improve rumen acidosis and fiber utilization for beef cattle that are fed with high-grain diets.

## Data Availability Statement

The datasets presented in this study can be found in online repositories. The name of the repository and accession number can be found below: National Center for Biotechnology Information (NCBI) BioProject, https://www.ncbi.nlm.nih.gov/bioproject/, PRJNA693324.

## Ethics Statement

The animal study was reviewed and approved by Lethbridge Research and Development Centre Institutional Animal Care and Use Committee (49).

## Author Contributions

WG, AS, TR, LJ, MS, EM, KO, LC, and WY: conceptualization, methodology, writing, reviewing, and editing. WG, AS, and LJ: formal analysis. WG: preparation and writing of the original draft. WY: supervision. WY and LC: project administration. WY, LC, EM, and KO: funding acquisition. All authors contributed to the article and approved the submitted version.

## Conflict of Interest

The authors declare that the research was conducted in the absence of any commercial or financial relationships that could be construed as a potential conflict of interest.

## References

[1] Isaak CK Petkau JC Karmin O Ominski K Rodriguez-Lecompte JC Siow YL. Seasonal variations in phenolic compounds and antioxidant capacity of *Cornus stolonifera* plant material: applications in agriculture. Can J Plant Sci. (2013) 93:725–34. 10.4141/cjps2012-310

[2] BenSaad LA Kim KH Quah CC Kim WR Shahimi M. Anti-inflammatory potential of ellagic acid, gallic acid and punicalagin A&B isolated from *Punica granatum*. BMC Complement Alter Med. (2017) 17:47. 10.1186/s12906-017-1555-0 28088220 PMC5237561

[3] Nair GG Nair CKK. Radioprotective effects of gallic acid in mice. Bio Med Res Int. (2013) 2013:953079. 10.1155/2013/953079 24069607 PMC3771270

[4] Kilic I Yeşiloglu Y Bayrak Y. Spectroscopic studies on the antioxidant activity of ellagic acid. Spectrochim Acta Part A Mol Biomol Spectrosc. (2014) 130:447–52. 10.1016/j.saa.2014.04.052 24813273

[5] Wei LY Jiao PX Alexander TW Yang WZ. Inclusion of Red osier dogwood in high-forage and high-grain diets affected *in vitro* rumen fermentation. Ann Anim Sci. (2018) 18:453–67. 10.1515/aoas-2017-0042

[6] Wei L Gomaa W Ametaj B Alexander T Yang W. Feeding red osier dogwood (*Cornus sericea*) to beef heifers fed a high-grain diet affected feed intake and total tract digestibility. Anim Feed Sci Technol. (2019) 247:83–91. 10.1016/j.anifeedsci.2018.11.006

[7] Spiehs M Varel V. Nutrient excretion and odorant production in manure from cattle fed corn wet distillers grains with solubles. J Anim Sci. (2009) 87:2977–84. 10.2527/jas.2008-1584 19502500

[8] Apea-Bah FB Head D Scales R Bazylo R Beta T. Hydrothermal extraction, a promising method for concentrating phenolic antioxidants from red osier dogwood (*Cornus stolonifer*) leaves and stems. Heliyon. (2020) 6:e05158. 10.1016/j.heliyon.2020.e05158 33083615 PMC7550924

[9] Yang W Gomaa W Saleem A McGeough E Ominski K Chen L. PSII-16 effect of red osier dogwood extract on *in vitro* digestibility and fermentation characteristics of high-grain diet. J Anim Sci. (2020) 98:403–4. 10.1093/jas/skaa278.708

[10] Czerkawski JW Breckenridge G. Design and development of a long term rumen simulation technique (Rusitec). Br J Nutr. (1977) 38:371–84. 10.1079/BJN19770102 588537

[11] McDougall E. Studies on ruminant saliva. 1. The composition and output of sheep's saliva. Biochem J. (1948) 43:99–109. 10.1042/bj0430099 16748377 PMC1274641

[12] Rhine E Mulvaney R Pratt E Sims G. Improving the Berthelot reaction for determining ammonium in soil extracts and water. Soil Sci Soc Am J. (1998) 62:473–80. 10.2136/sssaj1998.03615995006200020026x

[13] Winter KA Johnson R Dehority B. Metabolism of urea nitrogen by mixed cultures of rumen bacteria grown on cellulose. J Dairy Sci. (1964) 47:793–7. 10.3168/jds.S0022-0302(64)88766-X

[14] Li Y McAllister T Beauchemin K He M McKinnon J Yang W. Substitution of wheat dried distillers grains with solubles for barley grain or barley silage in feedlot cattle diets: intake, digestibility, and ruminal fermentation. J Anim Sci. (2011) 89:2491–501. 10.2527/jas.2010-3418 21454864

[15] AOAC. Association of Official Analytical Chemists. Official Methods of Analysis. 16th ed. Washington, DC: Association of Official Analytical Chemists (2006).

[16] Van Soest PV Robertson J Lewis B. Methods for dietary fiber, neutral detergent fiber, and nonstarch polysaccharides in relation to animal nutrition. J Dairy Sci. (1991) 74:3583–97. 10.3168/jds.S0022-0302(91)78551-2 1660498

[17] Rode LM Yang WZ Beauchemin KA. Fibrolytic enzyme supplements for dairy cows in early lactation. J Dairy Sci. (1999) 82:2121–26. 10.3168/jds.S0022-0302(99)75455-X 10531597

[18] Avila-Stagno J Chaves AV Ribeiro GO Jr Ungerfeld EM McAllister TA. Inclusion of glycerol in forage diets increases methane production in a rumen simulation technique system. Br J Nutr. (2014) 111:829–35. 10.1017/S0007114513003206 24094291

[19] Walters W Hyde ER Berg-Lyons D Ackermann G Humphrey G Parada A. Improved bacterial 16S rRNA gene (V4 and V4-5) and fungal internal transcribed spacer marker gene primers for microbial community surveys. Msystems. (2016) 1:e00009–15. 10.1128/mSystems.00009-15 27822518 PMC5069754

[20] Bolyen E Rideout JR Dillon MR Bokulich NA Abnet CC Al-Ghalith GA. Reproducible, interactive, scalable and extensible microbiome data science using QIIME 2. Nat Biotechnol. (2019) 37:852–7. 10.1038/s41587-019-0209-9 31341288 PMC7015180

[21] Love MI Huber W Anders S. Moderated estimation of fold change and dispersion for RNA-seq data with DESeq2. Genome Biol. (2014) 15:550. 10.1186/s13059-014-0550-8 25516281 PMC4302049

[22] Humer E Petri RM Aschenbach JR Bradford BJ Penner GB Tafaj M. Invited review: practical feeding management recommendations to mitigate the risk of subacute ruminal acidosis in dairy cattle. J Dairy Sci. (2018) 101:872–88. 10.3168/jds.2017-13191 29153519

[23] Russell JB Wilson DB. Why are ruminal cellulolytic bacteria unable to digest cellulose at low pH? J Dairy Sci. (1996) 79:1503–9. 10.3168/jds.S0022-0302(96)76510-4 8880476

[24] Penner G Beauchemin K Mutsvangwa T. Severity of ruminal acidosis in primiparous Holstein cows during the periparturient period. J Dairy Sci. (2007) 90:365–75. 10.3168/jds.S0022-0302(07)72638-3 17183105

[25] Morvay Y Bannink A France J Kebreab E Dijkstra J. Evaluation of models to predict the stoichiometry of volatile fatty acid profiles in rumen fluid of lactating Holstein cows. J Dairy Sci. (2011) 94:3063–80. 10.3168/jds.2010-3995 21605776

[26] Miśta S Pecka E Zachwieja A Zawadzki W Bodarski R Paczyńska K. *In vitro* ruminal fluid fermentation as influenced by corn-derived dried distillers' grains with solubles. Folia Biol. (2014) 62:345–51. 10.3409/fb62_4.345 25916162

[27] Tedeschi L Kononoff PJ Karges K Gibson M. Effects of chemical composition variation on the dynamics of ruminal fermentation and biological value of corn milling (co) products. J Dairy Sci. (2009) 92:401–13. 10.3168/jds.2008-1141 19109298

[28] Li Y He M Li C Forster R Beauchemin KA Yang W. Effects of wheat dried distillers' grains with solubles and cinnamaldehyde on *in vitro* fermentation and protein degradation using the Rusitec technique. Archiv Anim Nutr. (2012) 66:131–48. 10.1080/1745039X.2012.656479 22641925

[29] Beliveau RM McKinnon JJ. Effect of graded levels of wheat-based dried distillers' grains with solubles on rumen fermentation in finishing cattle. Can J Anim Sci. (2009) 89:513–20. 10.4141/CJAS08113

[30] Tedeschi LO Fox DG Russell JB. Accounting for ruminal deficiencies of nitrogen and branched-chain amino acids in the structure of the Cornell net carbohydrate and protein system. In: *Proceedings of Cornell Nutrition Conference for Feed Manufacturers*. New York, NY: Cornell University (2000). 10.2527/2000.7861648x

[31] Böttger C Südekum KH. European distillers dried grains with solubles (DDGS): chemical composition and *in vitro* evaluation of feeding value for ruminants. Anim Feed Sci Technol. (2017) 224:66–77. 10.1016/j.anifeedsci.2016.12.012

[32] Oba M. Effects of feeding sugars on productivity of lactating dairy cows. Can J Anim Sci. (2011) 91:37–46. 10.4141/CJAS10069

[33] He Z He M Walker N McAllister T Yang W. Using a fibrolytic enzyme in barley-based diets containing wheat dried distillers grains with solubles: ruminal fermentation, digestibility, and growth performance of feedlot steers. J Anim Sci. (2014) 92:3978–87. 10.2527/jas.2014-7707 24987082

[34] Nuez Ortín W Yu P. Nutrient variation and availability of wheat DDGS, corn DDGS and blend DDGS from bioethanol plants. J Sci Food Agric. (2009) 15:1754–61. 10.1002/jsfa.365220583193

[35] Lila ZA Mohammed N Kanda S Kamada T Itabashi H. Effect of sarsaponin on ruminal fermentation with particular reference to methane production *in vitro*. J Dairy Sci. (2003) 86:3330–6. 10.3168/jds.S0022-0302(03)73935-6 14594252

[36] Boadi D Benchaar C Chiquette J Masse D. Mitigation strategies to reduce enteric methane emissions from dairy cows: update review. Can J Anim Sci. (2004) 84:319–35. 10.4141/A03-109

[37] McGinn SM Chung YH Beauchemin KA Iwaasa AD Grainger C. Use of corn distillers' dried grains to reduce enteric methane loss from beef cattle. Can J Anim Sci. (2009) 89:409–13. 10.4141/CJAS08133

[38] Hristov AN Oh J Firkins JL Dijkstra J Kebreab E Waghorn G. Special topics–mitigation of methane and nitrous oxide emissions from animal operations: I. A review of enteric methane mitigation options. J Anim Sci. (2013) 91:5045–69. 10.2527/jas.2013-6583 24045497

[39] Dugan MER Aldai N Kramer JKG Gibb DJ Juárez M McAllister TA. Feeding wheat dried distillers grains with solubles improves beef trans and conjugated linoleic acid profiles. J Anim Sci. (2010) 88:1842–47. 10.2527/jas.2009-2575 20118430

[40] Jiao J Li X Beauchemin KA Tan Z Tang S Zhou C. Rumen development process in goats as affected by supplemental feeding v. grazing: age-related anatomic development, functional achievement and microbial colonisation. Br J Nutr. (2015) 113:888–900. 10.1017/S0007114514004413 25716279

[41] Bi Y Zeng S Zhang R Diao Q Tu Y. Effects of dietary energy levels on rumen bacterial community composition in Holstein heifers under the same forage to concentrate ratio condition. BMC Microbiol. (2018) 18:69. 10.1186/s12866-018-1213-9 29996759 PMC6042446

[42] Henderson G Cox F Ganesh S Jonker A Young W Abecia L. Rumen microbial community composition varies with diet and host, but a core microbiome is found across a wide geographical range. Sci Rep. (2015) 5:14567. 10.1038/srep14567 26449758 PMC4598811

[43] Callaway TR Dowd SE Edrington TS Anderson RC Krueger N Bauer N. Evaluation of bacterial diversity in the rumen and feces of cattle fed different levels of dried distillers grains plus solubles using bacterial tag-encoded FLX amplicon pyrosequencing. J Anim Sci. (2010) 88:3977–83. 10.2527/jas.2010-2900 20729286

[44] Zhang R Ye H Liu J Mao S. High-grain diets altered rumen fermentation and epithelial bacterial community and resulted in rumen epithelial injuries of goats. Appl Microbiol Biotechnol. (2017) 101:6981–92. 10.1007/s00253-017-8427-x 28762001

[45] Accetto T Avguštin G. The diverse and extensive plant polysaccharide degradative apparatuses of the rumen and hindgut Prevotella species: a factor in their ubiquity? Syst Appl Microbiol. (2019) 42:107–16. 10.1016/j.syapm.2018.10.001 30853065

[46] Singh RK Dey A Paul SS Singh M Dahiya SS Punia BS. Associative effects of plant secondary metabolites in modulating *in vitro* methanogenesis, volatile fatty acids production and fermentation of feed in buffalo (*Bubalus bubalis*). Agrofor Syst. (2020) 94:1555–66. 10.1007/s10457-019-00395-3

[47] Makkar H Francis G Becker K. Bioactivity of phytochemicals in some lesser-known plants and their effects and potential applications in livestock and aquaculture production systems. Animal. (2007) 1:1371–91. 10.1017/S1751731107000298 22444893

[48] Gomaa W Wei L Mosaad GM Aamer H Alexander TW Yang W. *In situ* ruminal digestibility of red osier dogwood in finishing beef heifers. Can J Anim Sci. (2018) 98:888–92. 10.1139/cjas-2017-0092

[49] Canadian Council on Animal Care. Guide to the Care Use of Farm Animals in Research Teaching Testing. In: Olfert ED, Cross BM, McWilliam AA, editors. Ottawa, ON: Canadian Council on Animal Care (2009).

